# Analyzing dendritic spine pathology in Alzheimer’s disease: problems and opportunities

**DOI:** 10.1007/s00401-015-1449-5

**Published:** 2015-06-11

**Authors:** Mario M. Dorostkar, Chengyu Zou, Lidia Blazquez-Llorca, Jochen Herms

**Affiliations:** Ludwig-Maximilians University Munich, Center for Neuropathology and Prion Research, Feodor-Lynen-Str. 23, 81377 Munich, Germany; Graduate School of Systemic Neuroscience, Ludwig-Maximilians-University Munich, Munich, Germany; German Center for Neurodegenerative Diseases (DZNE), Feodor-Lynen-Str. 23, 81377 Munich, Germany; Munich Cluster of Systems Neurology (SyNergy), Munich, Germany

## Abstract

Synaptic failure is an immediate cause of cognitive decline and memory dysfunction in Alzheimer’s disease. Dendritic spines are specialized structures on neuronal processes, on which excitatory synaptic contacts take place and the loss of dendritic spines directly correlates with the loss of synaptic function. Dendritic spines are readily accessible for both in vitro and in vivo experiments and have, therefore, been studied in great detail in Alzheimer’s disease mouse models. To date, a large number of different mechanisms have been proposed to cause dendritic spine dysfunction and loss in Alzheimer’s disease. For instance, amyloid beta fibrils, diffusible oligomers or the intracellular accumulation of amyloid beta have been found to alter the function and structure of dendritic spines by distinct mechanisms. Furthermore, tau hyperphosphorylation and microglia activation, which are thought to be consequences of amyloidosis in Alzheimer’s disease, may also contribute to spine loss. Lastly, genetic and therapeutic interventions employed to model the disease and elucidate its pathogenetic mechanisms in experimental animals may cause alterations of dendritic spines on their own. However, to date none of these mechanisms have been translated into successful therapeutic approaches for the human disease. Here, we critically review the most intensely studied mechanisms of spine loss in Alzheimer’s disease as well as the possible pitfalls inherent in the animal models of such a complex neurodegenerative disorder.

## Neuropathology of Alzheimer’s disease

In 1906, Alois Alzheimer examined the brain of a 54-year-old woman, who had died after a three-year course of severe cognitive impairment and memory loss. He noticed distinct histological alterations in the cortex, such as fibrillary tangles inside neurons and extracellular deposits of a substance unknown to him, which has later been identified as amyloid beta [4]. Quantification of these neuropathological alterations during autopsy is used today to assess whether an individual suffered from the disease now bearing Alzheimer’s name and how far the disease has progressed [26, 196]. These alterations are thought to be caused by an imbalance of amyloid beta production and its removal from the brain, causing the aggregation of characteristic fibrillar amyloid deposits. In turn, amyloid toxicity, which may be mediated by oligomeric intermediates and/or fibrillar amyloid beta, is thought to cause tau hyperphosphorylation and inflammatory changes as endogenous reactions to the presence of noxic stimuli. This pathogenic mechanism, which is essentially covered by the amyloid cascade hypothesis [80], is founded on numerous animal models which are genetically engineered to develop amyloid plaques. These animal models recapitulate some but not all the typical histologic alterations such as amyloidosis, synapse and neuron loss, tau hyperphosphorylation and inflammation. Another line of evidence is that humans with Down syndrome develop similar pathological changes as a result of the triplication of chromosome 21, on which the amyloid precursor protein (APP) is encoded [212]. Also, familial forms of Alzheimer’s disease are caused by mutations either in APP or in one of the two presenilin genes, which code for the enzymes processing APP to beta amyloid, ultimately leading to an overproduction of beta amyloid [175]. Lastly, one of the main risk factors of sporadic Alzheimer’s is homozygosity for the ε4 variant of the apolipoprotein E gene (ApoE4) [14], which causes reduced amyloid beta clearance [34]. While the amyloid cascade hypothesis is not without controversy [33], there is ample evidence that amyloid beta and hyperphosphorylated tau protein as well as the resultant inflammation may damage synaptic function.

## Dendritic spines

### Dendritic spine structure

Dendritic spines are the morphologic correlates of excitatory postsynapses. Morphologically, spines are specialized protrusions from a dendrite’s shaft, where neurons form synapses to receive and integrate information [69]. Typically, three different spine shapes are distinguished: Mushroom spines, which have a large head and a thin neck; stubby spines which have a large head but no discernible neck; and thin spines, which are slender, filopodia-like protrusions without a discernible head. A number of specialized synaptic proteins, including scaffolding proteins and ion channels, are clustered [136, 176] at dendritic spines. Spine size and morphology may reflect anatomical circumstances. For instance, longer spines may be observed in brain regions where target axons are located farther away from dendrites, such as in the reticular nucleus of the thalamus and in the gelatinous substance of the spinal cord dorsal horn [66]. Most importantly, however, dynamic alterations in spine morphology affect functional characteristics. For instance, increase in spine head size helps accommodate higher receptor numbers, while shortening and widening of spine necks decrease the electrical resistance of the spine neck, thereby leading to larger excitatory postsynaptic potentials [220].

### Role of dendritic spines in synaptic plasticity

**Fig. 1:**
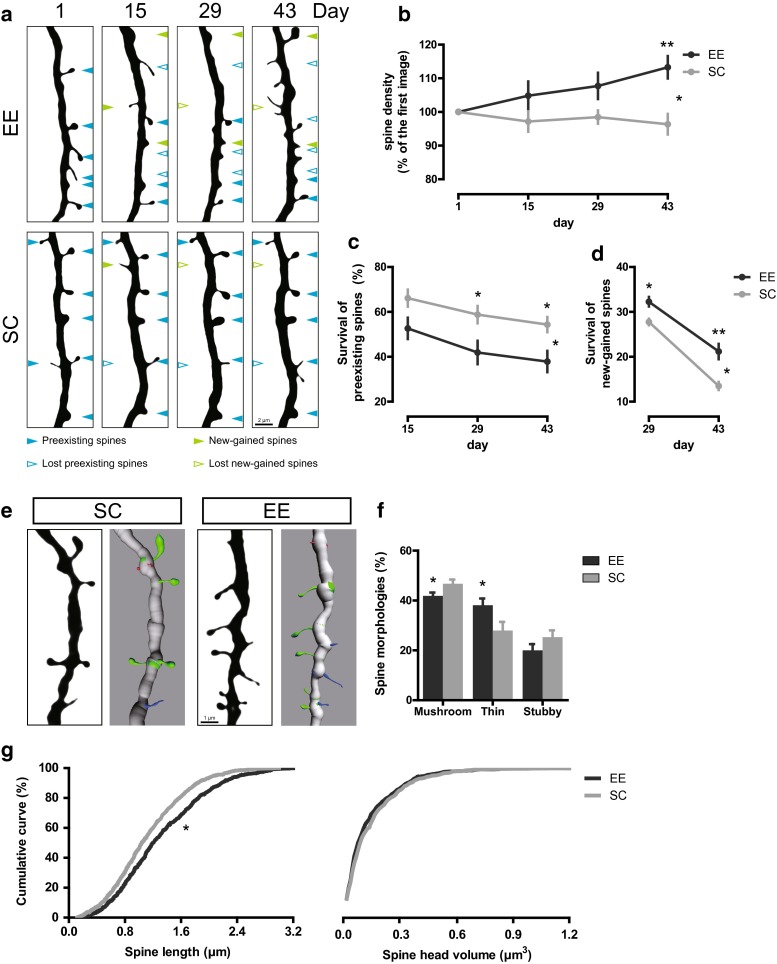
Dendritic spines are remodeled in enriched environment. **a** Chronic in vivo imaging of the same apical dendrites from layer V pyramidal neurons in the somatosensory cortex over 43 days when GFP-M mice were housed in standard conditions (SC) or enriched environment (EE). *Blue* and *green arrowheads* point to preexisting spines which are shown in the first image and new-gained spines that emerged over two consecutive imaging sessions, respectively. *Empty arrowheads* indicate the lost preexisting spines (*blue*) and new-gained spines (*green*). The images show high-contrast representations of maximum-intensity projected multiphoton images. **b** Graphical representations of the relative spine density. **c**, **d** The fate of preexisting spines in the first imaging time point and new-gained spines at the second imaging time point. **e** Apical dendrites (*black*) from layer V pyramidal neurons housed in SC or EE and 3D reconstructions (*gray*) generated in Imaris. The images show high-contrast representations of maximum-intensity projected confocal images. Mushroom, thin and stubby spines are colored in *green*, *blue* and *red*. **f** Plot of mushroom, thin and stubby spine fractions when mice were exposed to SC or EE. **g** Cumulative distributions of spine length and head volume when mice were exposed to SC or EE. *p < 0.05, **p < 0.01 (Two-way ANOVA in **b**–**d**, Student’s *t* test in **f** and Komogornov–Smirnov test in **g**)

Synaptic plasticity is in part mediated by altering the number of synaptic AMPA receptors through fast trafficking mechanisms [121]. However, these functional alterations are accompanied by morphological adaptations of dendritic spines, such as changes both in the number and shape of spines, which are termed structural plasticity [69] and are the focus of this review (Fig. 1). Such alterations have been observed to occur within minutes [108], yet they may also endure over longer time spans [218]. For instance, learning of motor tasks is associated with an increased spine formation [217] and a fraction of these newly formed spines may persist permanently [218]. Conversely, keeping animals in an enriched environment, which broadly stimulates motor, sensory and cognitive systems, increases the turnover, i.e., both the formation as well as the elimination of dendritic spines and this turnover. The net effect of these changes is an increased density of spines [93]. A key mediator for this effect is brain-derived neurotrophic factor (BDNF) [64], which acts via two receptors, NTRK2 (also known as TRKB) and NGFR (also known as p75NGF). A central regulator for structural plasticity is the enzyme glycogen synthase kinase 3β (GSK3β), which is a target of many psychotropic drugs [8]: Long-term potentiation, which is a functional correlate of synaptic plasticity, leads to inhibition of GSK3β [56, 146], which in turn increases structural plasticity by destabilization and increased turnover of dendritic spines [140]. Furthermore, a host of cytoskeletal proteins [178], as well as local protein translation [197], is required for the proper maintenance and turnover of dendritic spines.

### Dendritic spine pathology

**Fig. 2:**
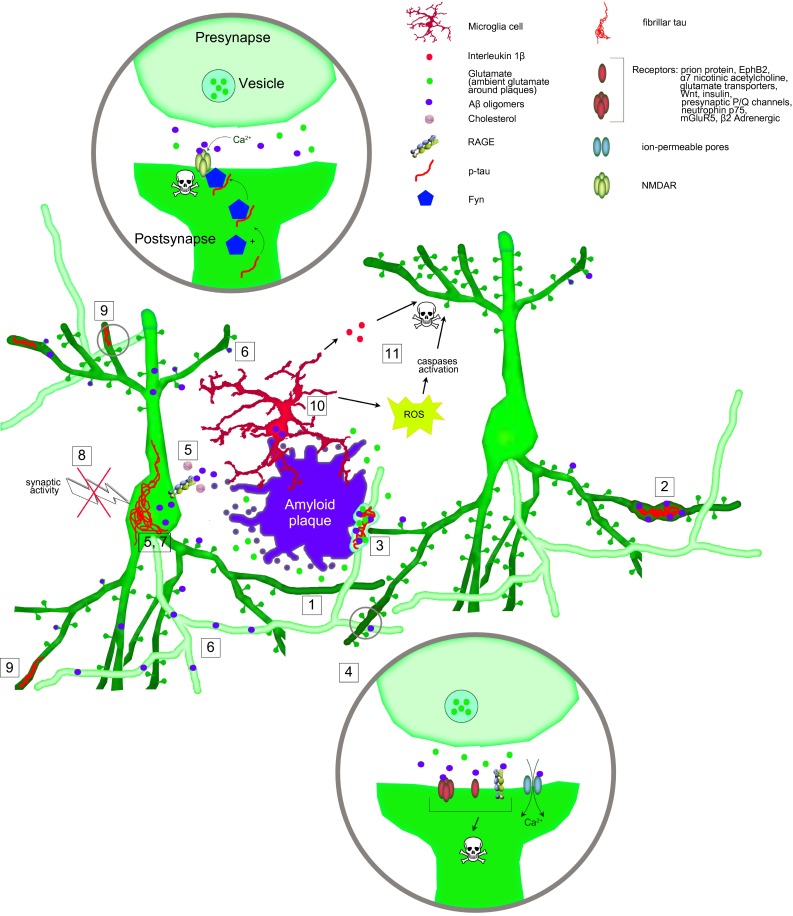
Putative pathophysiological mechanisms for dendritic spine loss. *1* Fibrillar amyloid plaques cause spine loss in their immediate vicinity. *2* Spine loss at dystrophic dendrites. *3* Secondary spine loss due to presynaptic failure. *4* Amyloid oligomers engaging synaptic targets. *5* Amyloid uptake. *6* Axonal amyloid beta. *7* Hyperphosphorylated tau protein. *8* Deafferentation. *9* NMDA receptor-mediated excitotoxicity. *10* Spine pruning by microglia; Microglia activation leading to release of inflammatory mediators. *11* Immune activation, reactive oxygen species and activation of pro-apoptotic pathways

Disturbance of the physiologic spine homeostasis underlies a number of neuropsychiatric disorders [147]. The most prominent example is loss of dendritic spines, which is encountered in most neurodegenerative disorders. Pathological spine loss can be caused by altering presynaptic input due to neuron-autonomous or extra-neuronal factors (Fig. 2). Synaptic factors for pathological spine loss may be deafferentation, which leads to a loss of complete dendrites [47, 90, 124], or sensory deprivation, which causes more complex changes: a retinal lesion, for instance, causes a complete replacement of spines in the deafferented cortex [95]. Pathological activation of NMDA receptors during excitotoxicity [78, 82] or disruption of dendritic transport both lead to spine loss and can be considered neuron-autonomous causes. Similarly, disruption of local protein synthesis at the spine may alter spine densities and morphology [199]. Examples for extraneuronally caused spine loss are trauma or inflammation, which in turn act through multiple mechanisms. Trauma causes initial spine loss mediated by calcineurin, followed by an overgrowth of spines [31]. Inflammation causes secretion of interleukin 1β, which antagonizes the action of BDNF, thereby leading to spine loss [201]. Tumor necrosis factor α (TNFα) from activated microglia leads to phosphorylation and upregulation of AMPA receptors, which in turn causes excitotoxicity [65, 109], thereby leading to spine loss [36]. Lastly, alterations in the composition of the extracellular matrix are associated with synapse loss [132].

On the other hand, not only spine loss, but also an increased stability or density of spines may be consequences of pathological mechanisms. Patients suffering from fragile X syndrome, which causes severe mental retardation, have elevated spine numbers [87]. Similarly, some neuropsychiatric diseases are accompanied with elevated spine densities in specific brain regions [147]. Thus, high spine densities per se are not necessarily desirable, either.

## Evidence for synapse loss in Alzheimer’s disease

A landmark study in the 1990s analyzed biopsies from individuals with clinically manifest Alzheimer’s disease using electron microscopy and correlated synapse numbers with results from the Mini-Mental Status examination, which is a measure for cognitive function: Patients with Alzheimer’s disease showed a significant loss of synapses compared with cognitively normal controls, and their cognitive capabilities correlated with synapse density [53, 195]. Follow-up studies on post-mortem tissue were able to analyze brain regions which are not amenable to biopsy. These showed, for instance, that individuals with early forms of Alzheimer’s disease had significantly fewer synapses in the inferior temporal gyrus, which plays an important role in verbal fluency [171], in the CA1 region [169], in the dentate gyrus [170] and in the posterior cingulate gyrus, which is a cortical region affected early during the onset of Alzheimer’s disease [168]. Immunohistochemical analyses showed loss of the presynaptic marker synaptophysin [123]. Moreover, a recent postmortem study using intracellular injections of Lucifer yellow in the brains of 5 Alzheimer’s disease patients revealed that intraneuronal tau aggregates are associated with a progressive alteration of dendritic spines [127].

Further evidence for the loss of synaptic function comes from in vivo PET imaging studies. These use radionuclide-labeled agonists for specific neurotransmitter receptors to measure the abundance of these receptors in various brain regions. One such study showed loss of α4β2 nicotinic acetylcholine receptors in the medial frontal cortex and nucleus basalis magnocellularis, which suggests loss of cholinergic synapses. This loss of α4β2 receptors correlated with increasing amyloid levels and with a loss of specific cognitive functions [141]. CB_1_ cannabinoid receptors, in contrast, were not altered [2] and 5HT_4_ serotonin receptors were increased with increased amyloid deposition [96, 118]. 5HT_1_ serotonin receptors were lost in late stages of Alzheimer’s disease [129]. These changes at the synaptic level have prominent effects on a more global scale, leading to paradoxical hyperexcitability and disruption of large-scale networks [76, 182, 183], which in turn are thought to be functional correlates of clinically apparent symptoms like impaired memory and cognition.

## Pathogenesis of dendritic spine loss in Alzheimer’s disease

Accumulation of amyloid beta is thought to be the initial causative factor leading to progressive synaptic injury [80]. However, secondary neuropathological alterations such as tau hyperphosphorylation or inflammation and consecutive dendritic and axonal dysfunction may cause synaptic damage on their own or exacerbate damage caused by amyloid beta.

### Amyloid beta

Amyloid beta is one of a multitude of enzymatic cleavage products of APP [135] and its secretion into the extracellular space is increased with neuronal activity [45, 94] through activation of extrasynaptic NMDA receptors [24]. To be more precise, however, amyloid beta does not refer to a singular chemical substance, but to several, depending on the exact cleavage sites and post-translational modifications including oxidation, phosphorylation, nitration, racemization, isomerization, pyroglutamylation, and glycosylation [103]. For instance, the 42 amino acid version (Aβ_1–42_) has a stronger propensity to aggregate than the 40 amino acid version (Aβ_1–40_). Modifications at the N-terminus further alter the protein’s biophysical properties. Of particular interest, pyroglutamate amyloid beta (Aβ_p3–42_), which has a cyclized glutamate residue at the N-terminus, has an even stronger propensity to aggregate [172] and seems to be specific for fibrillar plaques [54]. Amyloid beta can be detected in the extracellular as well as in the intracellular compartment, in oligomeric as well as fibrillar states. All these forms have been implicated in synaptic damage, which will be discussed in the following subsections.

## Amyloid beta plaques

**Fig. 3:**
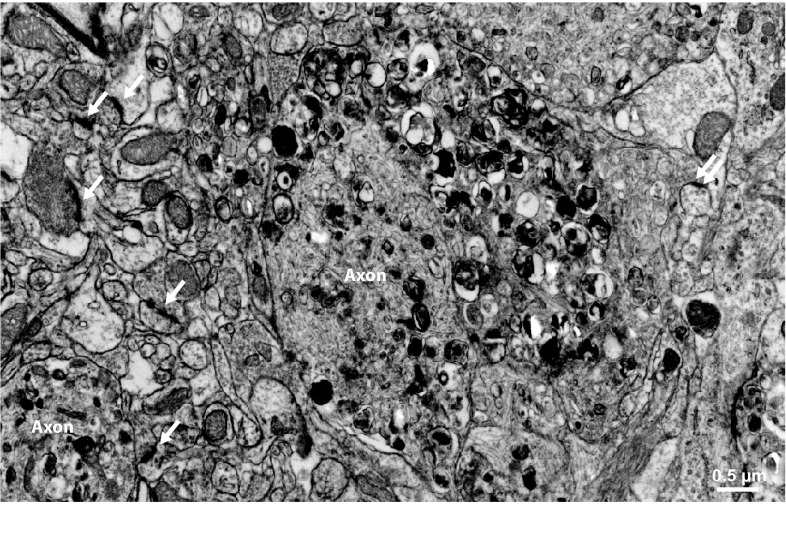
Neuritic pathology in a mouse model of amyloidosis. Electron micrograph of neuropil in the vicinity of a fibrillar plaque (not shown) of an APPPS1 mouse. Dystrophic axons (labeled “Axon”) appear enlarged and filled with electron-dense membranous material. Several synaptic densities are marked with *arrows*. Note that most synapses originate from unaltered synaptic boutons, while only a single synapse originates from the dystrophic axon (*double arrow*, far *right*), which may reflects spine loss as a result of presynaptic failure

**Fig. 4:**
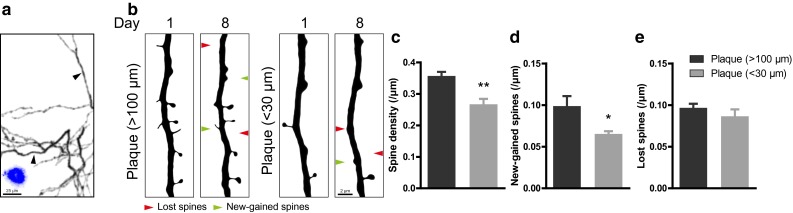
Spine loss is observed on dendrites that are close to amyloid plaques. **a** GFP-labeled dendrites (*black*) and methoxy-X04-labeled amyloid plaques (*blue*) from APPPS1xGFP-M mice. The images show high-contrast representations of maximum-intensity projected multiphoton images. *Black arrowheads* point to dendrites that are located close (<30 µm) to or far from (>100 µm) plaques, which are shown enlarged in **b**. **b** Maximum intensity projected apical dendrites that are located close (<30 µm) to or far from (>100 µm) amyloid plaques. The same dendrites were repeatedly imaged 1 week apart. *Red arrowheads* point at spines eliminated over 1 week and *green arrowheads* point at newly formed spines. **c** Spine densities of apical dendrites on different localizations (>100 or <30 µm from plaques). Spines that were new-gained **d** or lost **e** over 1 week on dendrites (>100 or <30 µm from amyloid plaques). *p < 0.05, **p < 0.01 (Student’s *t* test)

Amyloid plaques are the characteristic extracellular deposits of amyloid beta. Histologically, plaques may either appear either as diffuse plaques, which do not contain fibrils, and are detectable only in immunohistochemical stains using antibodies directed against APP epitopes. Alternatively, they may appear as cored plaques, which are composed of a fibrillar core and may be surrounded by a diffuse, non-fibrillar halo. The fibrillar core is detectable in H&E sections and can be stained with dyes specific for fibrillar aggregates such as Congo red or thioflavin S. Radiolabeled derivatives of these dyes, such as Pittsburgh compound B or florbetaben, are used as PET tracers to detect fibrillar amyloid beta in clinical settings, while fluorescent derivatives such as methoxy-X04 are used in animal studies for in vivo microscopy. As all of these compounds exclusively detect fibrillar protein aggregates, the majority of clinical and experimental in vivo studies have focused on fibrillar amyloid deposits. Indeed, there is ample evidence that fibrillar amyloid beta causes synaptic damage. In human cases, fibrillar amyloid plaques are typically surrounded by dystrophic neurites, which give rise to the so-called neuritic plaque appearance in silver stains or immunohistochemical stains against hyperphosphorylated tau protein using AT8 antibodies. In the human disease, dense neuritic AT8-staining also occurs distant to plaques in the form of neuropil threads, and the location and extent of this staining are the decisive measures to obtain the neuropathological staging according to the Braak and Braak criteria [26], which correlate best with the cognitive status. Some, but not all, mouse models of amyloidosis exhibit similar neuritic plaques, which are, like in the human disease, detectable using AT8 antibody staining or silver impregnation [39, 43, 154, 157, 184]. In contrast to the human disease, however, neuritic pathology in mouse models of amyloidosis is always limited to the immediate vicinity of fibrillar plaques (Fig. 3), while neuropil threads distant to plaques have not been observed. This might explain why in several animal models of Alzheimer’s disease which overexpress mutant human APP and/or presenilin alterations in spine density of layer 3 and 5 pyramidal neurons are apparent only in close vicinity of plaques (Fig. 4) [17, 97, 99, 214]. We found that spine loss occurred with a delay of at least 4 weeks after plaques had formed in one APP/PS1 mouse model [17]. However, the mechanisms leading to spine loss may differ between mouse models even if they share similar transgenes [228]. Nevertheless, in some mouse models, spine loss apparently independent of plaques was observed [19, 104]. In the triple transgenic mouse model co-expressing mutant APP, PS1 and tau, which we had analyzed, this spine loss occurred only at dystrophic dendrites with intracellular accumulation of both soluble amyloid beta and hyperphosphorylated tau protein [19]. Since substantial axonal damage occurs at amyloid plaques, secondary spine loss as a consequence of presynaptic failure [1] in those regions where damaged axons project to is very likely. Moreover, chronically altered synaptic input may affect the overall dendritic complexity and length in aged APP/PS1 mice while the dendritic spine density remains unaltered [179]. Both scenarios would explain a slight decrease in overall synapse density in some mouse models of amyloidosis in the absence of a significant reduction of the spine density of layer 3 and 5 neurons. Furthermore, functional alterations of neurons near plaques also point to a role of fibrillar amyloid beta in the damage of synaptic function, either direct or indirect [30]. Functional links between neuronal and synaptic dysfunction may be the disturbance of intracellular calcium dynamics [38, 100] or mitochondrial integrity [216]. Furthermore, perisomatic GABAergic terminals are lost close to plaques [70], which may also contribute to hyperexcitability and spine loss.

## Amyloid beta oligomers

Amyloid beta readily aggregates into soluble oligomers, which are still diffusible, in contrast to insoluble fibrils. As oligomers may exert effects distant to the place of their generation, they offer a convenient explanation for one of the major unsolved elements in the amyloid cascade hypothesis: the spatial separation of initial amyloid deposition and tau pathology in the brain. In the early stages of Alzheimer’s disease, amyloid beta accumulates preferentially in neocortical regions [196], while tau pathology typically starts in the brainstem and the transentorhinal region [26, 27]. Furthermore, the early stages of tau pathology often occur in individuals who do not show extracellular fibrillar amyloid deposits [27]. Similar to amyloid beta itself, amyloid oligomers are a highly heterogeneous group of chemicals, which are often classified according to their structural properties. The following variants are commonly distinguished [73, 77]: Protofibrils, which are beta-sheet containing intermediates of synthetic amyloid beta fibrillization; Annular assemblies and globulomers, which are both synthetic products; Amyloid beta-derived diffusible ligands, which are small, diffusible synthetic products; Aβ*56, which are endogenous products, found in APP transgenic mice and correspond to 12-mers; Secreted amyloid beta dimers and trimers, which are produced by cultured cells and are resistant to proteolytic degradation. It is important to note, however, that this heterogeneity not only reflects biological variation, but also technical variation in the methods to produce synthetic oligomers or to isolate them from biological tissues [12].

Nevertheless, there are several studies which point to functional relevance of oligomers in the human disease. For instance, in subjects with plaque pathology, the concentrations of amyloid oligomers were indicative of whether the subject had suffered from dementia [63] and synapse loss correlated with oligomer levels [98]. Dimers, trimers and Aβ*56 have been found in human subjects, with different associations to aging and Alzheimer’s disease [111]. Among these, Aβ*56 levels seem to correlate with synapse loss and the presence of tau oligomers [35, 111]. One animal study showed that removal of oligomers by genetically switching off their production led to cognitive improvement [68]. In another study, we showed that immunotherapy directed against globulomers abolished synapse loss distant from plaques, while in proximity to plaques synapse loss was attenuated only by a small degree [59], suggesting that plaque-associated pathology is not primarily mediated by oligomers. To assess the mechanism by which amyloid oligomers are synaptotoxic, oligomers are usually generated in vitro and then tested for their specific effects. However, a large variety of oligomers may be generated, depending on the exact experimental protocol used [89], and no single form of oligomers is accepted as the major contributor in Alzheimer’s disease [12]. Therefore, oligomers have been found to exert a wide variety of harmful effects on synapses [11]. Indeed, amyloid oligomers have been shown to bind preferentially to synapses [223]. There, they interact with a wide range of synaptic targets, such as prion protein [205], EphB2, RAGE, α_7_ nicotinic acetylcholine receptors, glutamate transporters [105], the Wnt receptor frizzled [120], insulin receptors, presynaptic P/Q channels, the neutrophin receptor p75 [150], mGluR5 [155, 204], β_2_ Adrenergic receptors [210], or calcineurin [214]. They may disrupt the neuritic cytoskeleton [223] and exacerbate neuronal activity-dependent DNA damage [165]. Finally, oligomers may also form ion-permeable pores in cell membranes [106], causing unregulated calcium entry and thereby leading to synaptic toxicity. It should be noted that most studies showing effects on the binding of synthetic amyloid oligomers to certain synaptic and extrasynaptic receptors lack control studies that guarantee specificity for the effects. Often equal concentrations’ monomers are used as controls, yet amyloid beta has a propensity to spontaneously aggregate to oligomers under various conditions in vitro [16, 83, 209]. Also scrambled peptides are used as controls. These are inappropriate controls for the specificity of an oligomeric protein complex, as scrambled peptides may lose their propensity to aggregate. So other either naturally occurring oligomeric proteins, such as tau, α-synuclein, gp120, PrP_106–126_ or synthetic oligomeric protein preparations [156], would be more appropriate to prove that the proposed effects of amyloid beta oligomers are truly specific for amyloid beta oligomers rather than oligomeric aggregates in general. Another commonly used control are fibrillar amyloid beta preparations which are observed to be less or not toxic to syanpses. The problem here is that the concentration of protein complexes injected into the brain is impossible to estimate [11, 12]. Thus, because of the complex biochemical behavior of amyloid oligomers, a single control peptide may not suffice to cover all possible unspecific effects. Furthermore, our in vivo studies show convincing evidence that fibrillar amyloid beta is highly toxic to synapses [17, 19, 59]. Amyloid deposition and evidence of fibrillar amyloid are time-dependent processes, not only in humans but also in animal models of Alzheimer’s disease. Although the exact age at which fibrillar deposits can be detected varies between models, most show an initial phase where no or only very few plaques are present. Several studies showed spine loss or synaptic dysfunction in these models before the appearance of plaques, which was generally interpreted as effects being mediated by soluble amyloid beta [29, 32]. Similarly, mouse models with the Osaka mutation, which generates oligomeric, but not fibrillar amyloid, develop synapse loss [200]. Additionally, if crossed with human tau expressing mice, these mice also develop tau pathology [206].

It is important to note, however, that the absence of plaques does not necessarily attribute causation to soluble amyloid beta, as intraneuronally accumulated amyloid beta or the genetic manipulations to obtain animal models may affect dendritic spines on their own (see below).

## Intraneuronal amyloid beta

**Fig. 5:**
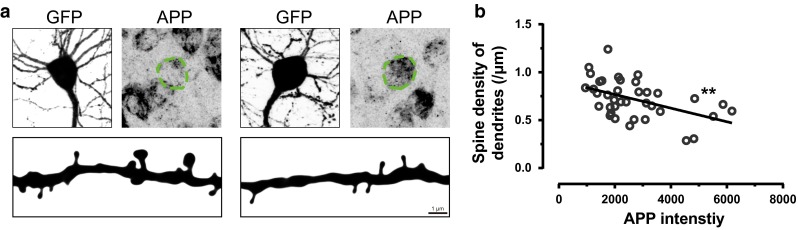
The content of intraneuronal APP is negatively correlated with spine density. **a** APP accumulation in GFP expressing layer V pyramidal neurons from APP23 mice (*top*). Dendrites that originated from the soma are shown below as high-contrast representations of maximum-intensity projected confocal images. **b** The *dot plot* is the corrected intensity of intercellular APP vs. spine density. *Straight line* is fitted by nonlinear regression. **p < 0.01 (*F* test). Figure adapted from Ref. [228]

Intraneuronal accumulation of amyloid beta has been observed in Down syndrome as well as in the early stages of Alzheimer’s disease [74, 125, 133]. However, in older individuals with Down syndrome and in late stages of Alzheimer’s disease, when abundant plaques are present in the brain, intraneuronal accumulation of amyloid beta is less evident [74, 133], although still present [138, 192]. While most studies suggested that intraneuronal amyloid beta is specific for disorders with extracellular amyloid deposition, one found intraneuronal amyloid beta was also in hippocampal neurons of control cases [20]. Conversely, a mouse model overexpressing APP with the Dutch mutation (E693Q) showed only intraneuronal amyloid accumulation, but no extracellular deposits [102]. Curiously, humans with this mutation suffer mainly from cerebral hemorrhage [112]. In animal models of amyloidosis, intraneuronal accumulation of amyloid beta increases with age [177, 192]. In one animal model, intraneuronal accumulation of APP was correlated with spine loss (Fig. 5) [228]. In another model, intraneuronal accumulation of oligomeric amyloid beta led to altered synapse structure in the hippocampus, while spine densities were not changed [151]. Furthermore, extracellular amyloid was found to be taken up into neurons via receptor for advanced glycosylation end products (RAGE) [193] and hypercholesterinemia accelerated uptake, leading to reduced synaptophysin immunoreactivity, and abnormal tau phosphorylation in the hippocampus [207]. The accumulation of amyloid in neurons also depended on the apolipoprotein E genotype, which is a known genetic risk factor for Alzheimer’s disease. The ε4 (ApoE4) isoform, which confers the highest risk for Alzheimer’s disease, also strongly increased the intraneuronal accumulation of amyloid [226].

Fibrillar amyloid was observed in dendrites close to plaques [122]. In an animal model with particularly strong pathology, the 5XFAD mouse model, fibrillar amyloid beta was detectable even in cell bodies [139], where it may have acted as seeds for plaques upon cell death [131]. Intracellular amyloid beta in axons, presynapses, as well as in dendritic spines was associated with synaptic pathology [44, 158, 190, 192]. Cytoplasmic amyloid beta was associated with mitochondrial alterations [158], apoptosis markers [61] and with oxidative damage to nucleic acids [138]. However, the latter study claimed that intracellular amyloid may be a compensatory mechanism, as amyloid beta has anti-oxidative properties [138]. Synaptic activity reduced intraneuronal amyloid and protected against amyloid-mediated synaptic alterations, while inhibition of synaptic activity increased intraneuronal amyloid and worsened synaptic damage [194]. It is, however, currently unclear whether intraneuronal amyloid beta contributes to synaptic damage in sporadic Alzheimer’s disease.

### Hyperphosphorylated tau protein

The deposition of hyperphosphorylated tau protein in neurons may be secondary to several different types of insults, such as epilepsy [134, 227], chronic traumatic brain injury [71], focal cortical dysplasia [174] or Niemann–Pick disease type C [117, 187]. Tau protein itself seems to be a prerequisite for neuronal damage, as tau knockout mice are immune to neuronal insults mediated by NMDA receptor-dependent excitotoxicity, as well as those caused by amyloid beta [110, 159, 160]. These findings corroborate tau pathology as a secondary effect of amyloid beta in Alzheimer’s disease. On the other hand, the degree of tau pathology is better correlated to cognitive decline than amyloid pathology [3, 13, 72]. Furthermore, a group of neurodegenerative diseases, collectively termed tauopathies, is characterized by mutations in tau protein, which lead to the deposition on hyperphosphorylated tau protein without an associated primary pathology. In experimental animals, the overexpression of wild-type human tau suffices for the formation of neurofibrillary tangles and age-dependent reductions in spine head volumes [57]. Similarly, the introduction of P301S mutant human tau causes inflammation and spine loss [10, 84]. These results suggest that hyperphosphorylated tau on its own may cause synaptic damage. Moreover, a recent study on 5 Alzheimer’s disease patients revealed a reduced spine number in distal parts of the dendritic tree in pyramidal neurons of the parahippocampal cortex and CA1 neurons with intraneuronal neurofibrillary tangles [127]. Another study found that loss of spinophilin-positive puncta in CA1 field and area 9, which are markers of dendritic spines, correlated with cognitive decline and tau pathology [3].

Physiologically, tau protein is primarily located in axons, where it is associated with the cytoskeleton. The physiological phosphorylation pattern of tau determines its subcellular localization: For instance, one specific phosphorylation pattern localizes tau to the nucleus, while another is required during mitosis [137]. LTP induction or pharmacological stimulation of synaptic activity increase translocation of tau to postsynapses [28]. Hyperphosphorylation of tau disrupts this localization pattern [22, 137, 222, 223]. Interestingly, there is also some evidence that phosphorylation of tau may be mediated by APP [137]. The subcellular localization of tau is also influenced by splicing [113] as well as by post-translational enzymatic cleavage [162]. In particular, truncation of tau by specific proteases may increase the toxicity of tau and facilitate hyperphosphorylation [225]. Different patterns of truncation and hyperphosphorylation give rise to different conformations, or “strains”, of tau, which underlie different tauopathies [166]. Furthermore, these tau strains can be propagated from human tissue to susceptible mouse models in a prion-like manner while maintaining their identity [46, 166]. A characteristic form of propagation of pathological tau has also been observed within the brain experimental animals. There, tau pathology spreads synaptically from neuron to neuron [52, 60, 114], which may be the mechanism underlying the specific spreading pattern of tau pathology in Alzheimer’s disease [25].

Tau has been claimed to mediate NMDA receptor-dependent excitotocixity via the Src-family tyrosine kinase Fyn [88] in a phosphorylation-dependent manner [130]. Dendritic tau localizes Fyn to dendrites, where it phosphorylates the GRIN2B subunit of NMDA receptors, thereby enhancing their function [164]. In mice lacking tau, less Fyn is present in dendrites, leading to lower GRIN2B phosphorylation [23]. Fyn, in turn, was found to be activated by oligomeric amyloid beta binding to the cellular prion protein [205]. Amyloid oligomers also disrupt the activity-dependent relocalization of tau to postsynapses [28] in a phosphorylation-dependent manner [128]. These studies provide causal links between the toxicity of amyloid oligomers and the physiological function of tau protein. Furthermore, tau deposits may also activate inflammatory processes, such as increased immunoreactivity for interleukin 1β and cyclooxygenase 2, which in turn activate microglia [10]. Alternatively, dendritic spine loss in Alzheimer’s disease may be simply the consequence of deafferentation and thus a secondary phenomenon that is not at all related to any pathological action of tau at the dendritic spine itself [127]. Hence, dendritic tau hyperphosphorylation and aggregation in sporadic Alzheimer’s disease may be a secondary or even compensatory phenomenon due to slowly progressing deafferentation/disconnection in the aging brain rather than the cause of dendritic spine loss or synaptic failure. The correlation between the detection of hyperphosphorylated tau and human aging is extraordinarily strong—probably stronger than the link to sporadic Alzheimer’s disease.

Similar to amyloid beta, hyperphosphorylated tau may form thioflavin-S binding, fibrillar aggregates which appear microscopically as neurofibrillary tangles, as well as soluble oligomers. In contrast to amyloid beta, however, the verdict seems to be clearer that neurofibrillary tangles themselves are functionally inert [84, 101, 167] and that soluble tau aggregates mediate synaptic damage [148, 219, 223]. Hyperphosphorylated tau was shown to localize to both pre- and postsynapses in multiple studies [81, 86, 88, 148, 188, 189, 191, 219], where it causes synaptic dysfunction by impairing the trafficking or synaptic anchoring [86] as well as the excitability [130]. In THY-Tau22 mice, which express tau with the G272V and P301S mutations, the synaptic enhancement induced by exogenous BDNF was lost due to impaired NMDA receptor function [28]. Electrophysiologically, synaptic dysfunction manifested as a presynaptic deficit in the probability of neurotransmitter release [86, 148] as well as altered excitability of neurons [50, 126, 161]. Our own in vivo imaging studies in P301S mice gave evidence for a postsynaptic accumulation of hyperphosphorylated tau only in spines of CA3 neurons but not within pyramidal neurons of the cerebral cortex [84]. Similar results were found in Alzheimer’s disease patients, where only the thorny excrescences of CA3 neurons, but not spines of cortical neurons, were found to contain hyperphosphorylated tau protein [21, 127]. This may be related to the fact that CA3 thorny excrescences may contain microtubules, whereas dendritic spines of cortical neurons have an actin-based cytoskeleton [42, 181].

### Inflammation

Both amyloid deposition and hyperphosphorylated tau lead to deposition of complement, activation of microglia, invasion of T-cells and release of pro-inflammatory cytokines [6, 10, 67, 116, 119, 198], which in turn may affect dendritic spines by multiple mechanisms. Inflammation also drives tau hyperphosphorylation and aggregation [15, 51], so that a detrimental positive-feedback loop may ensue. Fibrillar amyloid deposits are surrounded by proinflammatory complement complexes [116] and activated microglia which phagocytose protofibrillar amyloid. Increased micgroglial phagocytosis of amyloid attenuates amyloid deposition in animal models [107, 115]. On the other hand, amyloid-independent microglia activation, as it for example occurs in patients with multiple sclerosis or HIV, was found to have no relevant impact on the development of Alzheimer-associated cortical pathology [49, 142]. Furthermore, signs of inflammation have been observed in multiple animal models before plaques [79, 213, 219] or tangles [219] were present, which may suggest that inflammation—if it plays a causative role in sporadic Alzheimer diseases—plays a role early during the development of the disease. We, however, failed to observe significant microglia activation in transgenic amyloid mouse models prior to the occurrence of amyloid plaques [91].

The mechanisms by which inflammation affects dendritic spines include activation of caspases via reactive oxygen species released from inflammatory cells, which causes reductions in dendritic spines [48, 62, 149]. Furthermore, release of interleukin 1β by microglia during the inflammatory process affects dendritic spines by antagonizing the stimulatory effect of BDNF on spine genesis [201]. Microglia themselves also play a role in maintaining dendritic spines in the absence of inflammation by producing BDNF to stimulate spine growth [145] and by pruning of spines during development and plasticity [143, 202]. However, it is unclear whether these mechanisms also play a role in Alzheimer’s disease.

### Mechanisms independent of amyloid beta, tau and inflammation

Essentially all animal models of Alzheimer’s disease are mice engineered to express one or several of the proteins which are known to cause familial forms of amyloidosis or tauopathy in humans. However, the expression level of these artificially introduced proteins is manifold higher than naturally occurring levels, so that pathological alterations become apparent within the lifespan of the experimental animals. However, the overexpression of these proteins alone may cause alterations in dendritic spines directly or indirectly, which have to be taken into consideration before translating results from animal models to the human disease. This subsection summarizes the most important mechanisms by which overexpression of Alzheimer’s disease-related genes in mice alters dendritic spines.

In animal models of Down syndrome, which are trisomic for the APP gene locus and thus overexpress APP, spine loss and synaptic damage have been described [9, 208]. These may be the consequence of intraneuronal APP accumulation or the accumulation of BACE1 derived cleavage products rather than due to soluble amyloid species of any type or location [75]. Recently, knock-in animal models expressing physiological quantities of mutant APP have been generated to help differentiate between synaptic and cognitive effects caused by overexpression and those by mutation of APP. Interestingly, only the combination of several mutations caused cognitive deficits [163]. Furthermore, in many models of Alzheimer’s disease, overexpression of mutant human APP is often combined with overexpression of mutant human presenilin 1 (PS1), which speeds up amyloidosis in double transgenic mice, compared to single APP transgenes. In contrast to humans, however, the expression of mutant PS1 alone does not cause an amyloidosis in rodents—a phenomenon that is not well understood, but points to one out of several shortcomings of mouse models in Alzheimer’s disease research. Furthermore, we and others have demonstrated that overexpression of both wild-type and mutated human PS1 actually causes an increase in spine density in young transgenic animals [5, 92, 186]. Consistent with these findings, electrophysiological studies of different mouse lines overexpressing mutant PS1 showed significantly enhanced LTP at hippocampal synapses [5, 7, 55, 144, 173, 211, 221]. Furthermore, a recent study showed that knock-in of L435F mutated PS1, which is a loss-of-function mutation, on a PS2 knockout background led to reduced LTP in comparison to PS2 knockouts with wild type PS1 [215]. These effects are clearly independent of any synaptotoxic effects of amyloid beta, since amyloid beta is not enhanced in these mouse models. Rather, several reports point to an altered calcium homeostasis as underlying mechanism for disturbed dendritic spine plasticity, as presenilin mutations seem to interfere with physiological calcium release from intracellular stores. Different molecular mechanisms have been proposed, ranging from ER leak channel activity of PS1 itself [203, 224] to increased gating probabilities of IP3 receptors [40, 41], elevated expression of ryanodine receptors [37, 180, 185] and most recently to reduced synaptic STIM2 expression and impaired store-operated calcium entry [186]. As an indicator of modified calcium homeostasis, we confirmed upregulated RyR levels in A246E-PS1 overexpressing cortical neurons [92].Given the prominent role of dendritic calcium signalling in dendritic spine plasticity, we, therefore, favour the view that PS1-dependent changes in calcium homeostasis underlie the elevated spine densities in PS1-transgenic mice. It is still a matter of debate how the overexpression of PS1 affects the calcium homeostasis. Based on our cell culture studies and biochemical studies on postmortem brains of patients carrying familial Alzheimer’s disease PS1 mutations, we favour the notion that the disturbed ER calcium homeostasis is mediated by the elevation of PS1 holoprotein levels [85] possibly as a consequence of altered presenilin autocleavage. This hypothesis may have impact on the translation of therapeutic efforts from these familiar forms of Alzheimer’s disease, which are currently used to study the treatment of very early stages of Alzheimer’s disease like the Alzheimer’s Prevention Initiative enrolling members of a Columbian cohort who carry the E280A PS1 mutation, to the treatment of sporadic Alzheimer’s disease.

Presenilins are important constituents of the γ-secretase complex, which is necessary to generate amyloid beta, and hence also amyloid oligomers, from APP. Inhibitors of γ-secretase are, therefore, often used as a research tool to reduce the levels of amyloid beta oligomers in experimental animals and prove putative oligomer-dependent mechanisms. Inhibition of γ-secretase, however, leads to alterations in dendritic spines even in the absence of amyloidosis-related transgenes: By performing chronic in vivo two photon imaging in wild-type mice, we observed reduced spine densities after pharmacological inhibition of γ-secretase for 4 days [18]. This observation is in contradiction to ex vivo studies performed in cell culture or in organotypic cultures by various laboratories including our own where γ-secretase inhibition is used as a tool to inhibit amyloid beta production. There, γ-secretase inhibition had no acute effect on dendrites, spine morphology or excitatory synaptic transmission [152, 153, 221]. However, the duration of γ-secretase inhibition might be critical in order for these detrimental effects on spine plasticity to take effect. These preclinical in vivo findings in rodents might be relevant in the development of Alzheimer’s disease therapies aimed at interfering with the function of the γ-secretase to reduce the production of amyloid beta peptides. Our observation of reducing dendritic spine numbers in vivo following γ-secretase inhibition might offer a potential explanation why Alzheimer’s disease patients treated with a potent γ-secretase inhibitor (semagacestat) showed, among other side effects, a worsening of cognition in the high dose cohort, which caused a phase 3 study to be halted [58].

Because of the complex interplay between the physiological roles of proteins involved in Alzheimer’s disease, it is hard to differentiate disease-specific and hence therapeutically relevant effects from those which are related to the genetic manipulation of experimental models in the first place. This fact may, however, explain why such a large number of treatments which were effective in experimental animals have failed to yield any therapeutic benefit in humans.

## Conclusions

Loss of dendritic spines in Alzheimer’s disease is intimately linked with synaptic dysfunction and loss of memory and cognition—the very functions which define a human being. Understanding the mechanisms of synapse loss may enable us to find an appropriate therapy to halt or even reverse the progress of this debilitating disease. Unfortunately, the scientific findings to date suggest that an extremely complex pathophysiology underlies Alzheimer’s disease with a wide variety of possible mechanisms which may cause synapse loss or dysfunction. At the moment, it is unclear which of the mechanisms covered here (or indeed any of the multitude of mechanisms which we have not covered) is dominantly responsible for synapse dysfunction in human patients. To paraphrase Alzheimer’s own conclusion of his report, “On a peculiar disease of the cerebral cortex” [4]: We are obviously dealing with a peculiar disease process here. These observations should compel us not to content ourselves with forcibly applying the knowledge we have to date to explain insufficiently understood mechanisms. Future study will enable us to gradually untangle specific mechanisms and assess their contribution to the disease.
